# Hospitalization and emergency department visits among seniors receiving homecare: a pilot study

**DOI:** 10.1186/1471-2318-5-9

**Published:** 2005-07-13

**Authors:** Andrew A Smith, Soo B Chan Carusone, Kathleen Willison, Tamara J Babineau, Stephanie D Smith, Tom Abernathy, Tom Marrie, Mark Loeb

**Affiliations:** 1 Department of Pathology and Molecular Medicine, McMaster University, Hamilton, Canada; 2 Department of Clinical Epidemiology and Biostatistics, McMaster University, Hamilton, Canada; 3 Victorian Order of Nurses, Hamilton, Canada; 4 Central West Health Planning Information Network, Hamilton, Canada; 5 Department of Medicine Division of Infectious Diseases, University of Alberta, Edmonton, Canada; 6 Hamilton Regional Laboratory Medicine Program, Hamilton, Canada

## Abstract

**Background:**

Despite the recent growth in home health services, data on clinical outcomes and acute health care utilization among older adults receiving homecare services are sparse. Obtaining such data is particularly relevant in Ontario where an increasing number of frail seniors receiving homecare are awaiting placement in long-term care facilities. In order to determine the feasibility of a large-scale study, we conducted a pilot study to assess utilization of acute health care services among seniors receiving homecare to determine associated clinical outcomes.

**Methods:**

This prospective cohort study followed forty-seven seniors admitted to homecare by two homecare agencies in Hamilton, Ontario over a 12-month period. Demographic information and medical history were collected at baseline, and patients were followed until either termination of homecare services, death, or end of study. The primary outcome was hospitalization. Secondary outcomes included emergency department visits that did not result in hospitalization and death. Rates of hospitalization and emergency department visits without admission were calculated, and univariate analyses were performed to test for potential risk factors. Survival curves for accumulative rates of hospitalization and emergency department visits were created.

**Results:**

312 seniors were eligible for the study, of which 123 (39%) agreed to participate initially. After communicating with the research nurse, of the 123 who agreed to participate initially, 47 (38%) were enrolled in the study. Eleven seniors were hospitalized during 3,660 days of follow-up for a rate of 3.0 incident hospitalizations per 1,000 homecare-days. Eleven seniors had emergency department visits that did not result in hospitalization, for a rate of 3.3 incident emergency department visits per 1,000 homecare-days. There were no factors significantly associated with hospitalization or emergency department visits when adjustment was made for multiple comparisons.

**Conclusion:**

The incidence of hospitalization and visits to the emergency department among seniors receiving homecare services is high. Getting satisfactory levels of enrolment will be a major challenge for larger prospective studies.

## Background

Home health care is a rapidly growing health care sector [1]. An increasing older population and the desire to reduce acute health care costs have contributed to its growth. In the United States, the 2003 annual expenditure for home health care was estimated to be $38.3 billion. This includes approximately 20,000 providers delivering care to 7.6 million individuals [2]. In Canada, homecare expenditures in 2001 totalled approximately $3.1 billion accounting for 3.3% of total health care expenditures [3].

Homecare clients are disproportionately older, live alone, and are low income [4]. However, despite the recent growth in home health services, data on clinical outcomes and acute health care utilization among older adults receiving homecare services are sparse. Obtaining such data is particularly relevant in Ontario where an increasing number of frail seniors receiving homecare are awaiting placement in long-term care facilities. In order to determine the feasibility of a large-scale study, we conducted a pilot study to assess utilization of acute health care services among seniors receiving homecare to determine associated clinical outcomes.

## Methods

We conducted our study in Hamilton, Ontario, Canada, a city of approximately 490,000 people, where it is estimated by 2003 Canadian census that there are 70,000 people over the age of 65. Between October 2002 and October 2003, we enrolled individuals aged 65 years and over who were admitted to nursing homecare services provided by either one of two homecare agencies. Because we could not contact potential participants directly due to confidentiality, a volunteer at each of the two agencies would notify potentially eligible seniors about the study and would forward the list of any seniors who were willing to be approached.

In order to collect data on clinical outcomes, we limited enrolment to seniors in whom it was anticipated that homecare services would be provided for more than two months. Individuals who were expected to die within 30 days of recruitment were excluded. All seniors or their substitute decision makers were approached regarding the study, and informed consent was obtained for each participant. All participating homecare providers approved the study.

### Baseline assessment

Baseline demographic and clinical data were obtained at study entry for each senior enrolled. We recorded basic demographic information including age, sex, socioeconomic status, as well as the following other potential risk factors for hospitalization: use of medications, functional status, co-existing illnesses, receipt of influenza and pneumococcal vaccines, body mass index (weight in kilograms divided by height in meters^2^), nutritional status, and smoking and alcohol history. This information was obtained through interviews with the participants as well as through review of medical charts. We used the Charlson Index to quantify comorbid illness [5]. This method assigns weights to 19 categories of comorbidities based on the adjusted risk of one-year mortality (the higher the weight the more severe the burden). The Charlson Index score for an individual is calculated by summing the assigned weights. Functional status was assessed at study entry using the Barthel Index [6]. The Barthel index is a simple 10-item measure of independence based upon the ability of an individual to care for oneself. The values assigned to each item are based on the amount of assistance required for the individual to perform the activity (the maximum value is achieved only if no assistance is required). The Mini Nutritional Assessment (MNA) was used to determine nutritional status. This 10-item tool, designed to evaluate the risk of malnutrition, was developed and validated by the Centre for International Medicine and Clinical Gerontology of Toulouse, the Clinical Nutritional Program at the University of New Mexico, and the Nestlé Research Centre in Lausanne [7].

Each senior in the study was re-assessed by a research nurse after the first three months of follow-up to ensure that the information at baseline had not changed. This assured up-to-date information when assessing modifiable risk factors, such as immunization with the influenza or pneumococcal vaccine, or development of aspiration due to stroke.

### Outcome assessment

The main outcome of this study was hospitalization and emergency department visits that did not result in hospitalization. Seniors from the two homecare providers were observed until either the termination of their homecare or October 31, 2003, the end date of the study. The research nurse conducted telephone interviews with enrolled seniors biweekly to determine current homecare status and to record any changes to potential risk factors for hospitalization such as newly diagnosed conditions. Medical records of seniors who had hospitalizations and emergency department visits were reviewed.

### Statistical analysis

Using the number of days each senior was followed and the number of seniors followed over the study period, we calculated rates of hospitalization and emergency department visits without admission, expressed as the number of incident events per 1000 senior-homecare days. Kaplan-Meier survival estimates were constructed to determine cumulative incidence curves (including 95% confidence intervals). Seniors who were still receiving homecare at the study endpoint and who did not visit the emergency department during follow-up were censored. Chi-square tests were performed for dichotomous variables and student's t-test for continuous variables. We used a Bonferroni adjustment to adjust for multiple comparisons. Since there were 16 comparisons in each set of analyses (hospital admission and emergency department visit) we set the significance level at 0.05/16 or 0.003. Data entry and analysis were performed using commercially available software (SAS, version 8.02, SAS Institute, Inc., Cary, N.C.).

## Results

A total of 312 seniors were eligible for the study, of which 123 (39%) agreed to participate initially. After communicating with the research nurse, of the 123 who agreed to participate initially, 47 (38%) were enrolled in the study.

### Characteristics of study participants

The mean age of the 47 seniors enrolled in this study was 77.8 years (range 67 to 97 years) and 25 (53.2%) were female (Table 1). The mean body mass index score was 33.1 (range 11 to 77). Average scores for the Charlson Index was 1.70 (range 0 to 8), Barthel Index was 18.70 (range 11 to 20) and Nutrition Score was 3.98 (range 1 to 9). Thirty-three of the 47 participants lived in a house, 9 in an apartment, and 5 in a condominium. Twenty-eight were married, 16 widowed and the remaining 3 had never been married. Twenty-five (53%) completed high school or higher education.

**Table 1:** Demographic information of participants.

Patient demographic		Value	N
Age (Years)	mean (SD)	77.8 (6.5)	47
	range	67–97 years	
Sex – male	n (%)	22 (47%)	47
Body mass index score	mean (SD)	33.1 (14.2)	47
	range	11–77	
Charlson Index score	mean (SD)	1.70 (1.92)	47
	range	0–8	
Barthel Index score	mean (SD)	18.70 (1.99)	47
	range	11–20	
Nutrition score	mean (SD)	3.98 (2.46)	47
	range	1–9	
Residence	n (%)		47
House		33 (70%)	
Apartment		9 (19%)	
Condominium		5 (11%)	
Marital Status	n (%)		47
Married		28 (60%)	
Widowed		16 (34%)	
Single, never married		3 (6%)	
Education	n (%)		47
Completed high school or higher education		25 (53%)	

### Outcomes

There were a total of 4260 senior homecare-days of follow-up. The average follow-up duration was 90.6 days (range from 4 to 347 days); the median follow-up duration was 54.0 days. There were 11 seniors who were hospitalized during 3668 homecare-days of surveillance. There were 3.0 incident hospitalizations per 1000 homecare-days.

A total of 11 incident emergency department visits occurred during 3328 homecare-days of surveillance. There were 3.3 incident emergency department visits per 1000 homecare-days.

**Figure 1:**
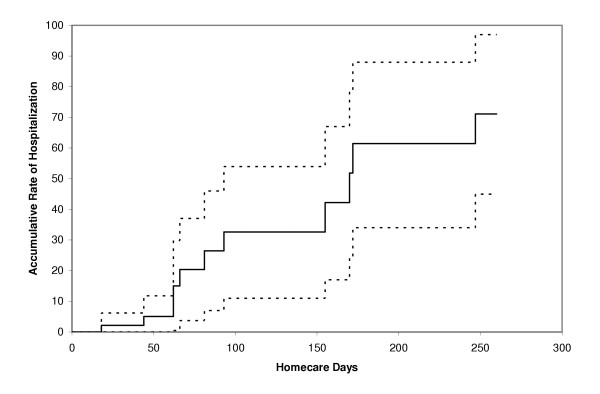
Kaplan-Meier curve of hospitalization among 47 seniors receiving homecare services.

Of the 11 seniors who were hospitalized, two were admitted for each of the following: congestive heart failure, dehydration, and pneumonia. One senior with each of the following diagnoses was admitted: anaphylactic reaction, pulmonary embolus, refractory vomiting, cellulitis, and excessive bleeding after pleural biopsy. The last two seniors were admitted a second time for accidental overdose and bowel obstruction respectively. The Kaplan-Meier curve for hospitalization is shown in Figure 1.

Of the eleven seniors seen in the emergency department but not admitted, one senior was admitted with each of the following: scalp laceration, soft tissue foot infection, urinary tract infection, urinary retention, bleeding mouth ulcer, alcohol intoxication, thrombocytopenia, and small bowel obstruction. The latter senior was admitted to the emergency department a second time with Enterococcal bacteremia. The senior seen for alcohol intoxication was admitted a second time for hyponatremia, hypophosphatemia and hypomagnesemia caused by alcohol and metformin induced diarrhea. The senior admitted with thrombocytopenia was admitted a second time for complications following a liver biopsy. The remaining three seniors were seen for cellulitis, one of whom visited the emergency department six more times for cellulitis without hospitalization and was finally hospitalized for cellulitis.

**Figure 2:**
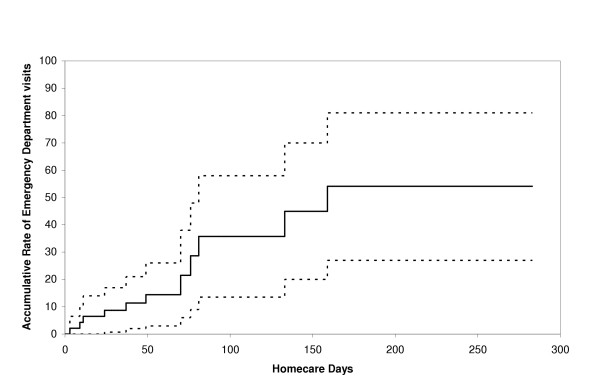
Kaplan-Meier curve of emergency department visits without hospitalization among 47 seniors receiving homecare services.

There were three emergency department visits followed by hospitalization. Two of these visits were attributed to shortness of breath, one caused by bilateral pneumonia and the other by congestive heart failure with a large right pleural effusion. The third patient visit was attributed to dehydration, malnutrition, nausea and anorexia secondary to bacillus *Clostridium *overgrowth resulting from antibiotic use. The Kaplan-Meier curve for emergency department visits is shown in Figure 2.

Five patients died after their first hospitalization and one patient died after a second hospitalization while participating in the study. Causes of death included respiratory failure, hypoxia after liver biopsy, bronchogenic carcinoma and congestive heart failure in two patients. Bowel obstruction was the cause of death for the patient who died after second hospitalization.

### Risk factors for hospitalization and emergency department visits

Risk factors for hospitalization and emergency department visits are summarized in Tables 2 and 3 respectively. Using a level of significance of 0.003, there were no significant risk factors for either outcome.

**Table 2:** Risk factors for hospitalization among a cohort of seniors admitted to homecare services

Potential risk factor	Seniors hospitalized (n = 11)	Seniors not hospitalized (n = 36)	Odds Ratio (95% CI)	P-value
Mean age in years (SD*)	78.2 (8.1)	77.6 (6.2)	1.01 (0.92, 1.12)	0.822
Male sex	8/11	14/36	4.19 (0.95, 18.53)	0.083
Diagnosed w/ pneumonia (in past 6 months)	1/11	6/36	0.50 (0.05, 4.67)	1.000
Influenza vaccine in last year	10/11	30/36	2.00 (0.21, 18.69)	1.000
Pneumococcal vaccine	7/10	21/35	1.56 (0.34, 7.06)	0.719
Swallowing difficulties	1/11	6/36	0.50 (0.05, 4.67)	1.000
Smoke	0/11	3/36	0	1.000
Consume alcohol	5/11	20/36	0.67 (0.17, 2.59)	0.732
Congestive Heart Failure	3/11	2/36	6.38 (0.91, 44.71)	0.076
Cerebrovascular disease	1/11	7/36	0.41 (0.05, 3.80)	0.659
Neoplastic disease	5/11	6/36	4.17 (0.95, 18.22)	0.097
Renal disease	3/11	0/36	8	0.010
Liver disease	2/11	2/36	3.78 (0.47, 30.64)	0.229
Charlson score^1^, mean (SD*)	3.27 (2.69)	1.22 (1.33)	1.77 (1.15, 2.71)	0.032
Nutrition Score^2^, mean (SD*)	5.82 (2.23)	3.42 (2.27)	1.51 (1.11, 2.06)	0.006
Barthel score^3^, mean (SD*)	18.36 (2.11)	18.81 (1.97)	0.90 (0.66, 1.24)	0.546

* SD: standard deviation

^1^Charlson Index: range 0 to 37

^2^Mini Nutritional Status Score: range 0 to 21

^3^Modified Barthel Index: range 0 to 20

**Table 3:** Risk factors for an emergency department visit not followed by hospitalization among a cohort of seniors receiving homecare services

Potential risk factor	Seniors with an emergency department visit (n = 11)	Seniors without an emergency department visit (n = 36)	Odds Ratio (95% CI)	P-value
Mean age in years (SD*)	76.98 (5.73)	78.00 (6.83)	0.98 (0.88, 1.09)	0.626
Male sex	6/11	16/36	1.50 (0.39, 5.83)	0.732
Diagnosed w/ pneumonia (in past 6 months)	2 /11	5/36	1.38 (0.23, 8.33)	0.659
Influenza vaccine in last year	10/11	30/36	2.00 (0.21, 18.69)	1.000
Pneumococcal vaccine	9/10	19/35	7.58 (0.87, 66.40)	0.064
Swallowing difficulties	1/11	6/36	0.50 (0.05, 4.67)	1.000
Smoke	0/11	3/36	0	1.000
Consume alcohol	3/11	22/36	0.24 (0.05, 1.06)	0.083
Congestive Heart Failure	3/11	2/36	6.38 (0.91, 44.71)	0.076
Cerebrovasucular disease	3/11	5/36	2.33 (0.46, 11.85)	0.367
Neoplastic disease	4/11	7/36	2.37 (0.54, 10.40)	0.256
Renal disease	2/11	1/36	7.78 (0.63, 95.68)	0.132
Liver disease	2/11	2/36	3.78 (0.47, 30.64)	0.229
Charlson score^1^, mean (SD*)	2.91 (2.02)	1.33 (1.76)	1.50 (1.04, 2.17)	0.034
Nutrition Score^2^, mean (SD*)	5.45 (2.73)	3.53 (2.22)	1.379 (1.032, 1.842)	0.051
Barthel score^3^, mean (SD*)	18.45 (1.97)	18.78 (2.02)	0.93 (0.67, 1.28)	0.642

* SD: standard deviation

^1^Charlson Index: range 0 to 37

^2^Mini Nutritional Status Score: range 0 to 21

^3^Modified Barthel Index: range 0 to 20

## Discussion

Little is known about the pattern of utilization of acute health care services among seniors receiving homecare. We found a high rate of use of acute health care services in this pilot cohort and identified comorbidity as a potentially important risk factor. These findings are particularly relevant given the aging population in many North American communities and the shortage of acute and long-term care beds. In addition to the increase in demand for homecare services, these findings suggest that the seniors receiving homecare in these communities may also utilize more acute health care services. If all seniors in this cohort had been followed for over eight months, a majority of them would have been hospitalized or had a visit to the emergency department (70% and 50% respectively). In comparison, an observational cohort study that followed 1,291 seniors receiving home health care in Italy reported a hospitalization rate of only 26% over a 12-month period [8]. These findings also suggest that acute health services utilization is significantly higher in homecare recipients than non-institutionalized community dwelling seniors in the same population. A cross-sectional study (also done in Ontario, Canada) of 1,038 seniors, reported a hospitalization rate of 17% over a 12-month period [9]. This stresses the need for a greater expertise in geriatrics among health care professionals working in emergency departments. For example, there may be an important role for geriatric nurse practitioners given the specific needs required in the assessment and follow-up of seniors.

Although statistical significance was not reached for individual potential risk factors, these pilot data are consistent with previous research findings and suggest that some factors associated with rehospitalization of seniors and acute health care utilization in seniors admitted to homecare are likely common, such as multiple comorbidities. Most of the existing homecare literature is either retrospective or descriptive in nature [10-13].

The determination of risk factors for acute care utilization in this population may enable more accurate identification of high-risk patients and assist in care planning and the appropriate use of targeted efforts. In contrast to the lack of homecare literature, numerous studies have been conducted to identify predictors of rehospitalization, as described in the review by Anderson and colleagues [10]. Prior hospitalizations, presence of a chronic disease, and severity of illness have consistently been identified as important predictors of rehospitalization, but many other determinants including social factors and circumstances associated with the process of care and discharge have also been assessed and found to be important in some studies [14-19].

There were a number of challenges when implementing this study. The first was determining the best way to enrol participants. We had initially anticipated enrolling individuals discharged to homecare from acute care hospitals. However, prior to beginning the study it became evident that we would not have cooperation of hospital case-managers, which meant that the research nurse would not be informed about potential participants. Therefore, we decided to enrol participants directly from homecare agencies within the community. The major difficulty, as can be seen by the low number of potentially eligible participants who were enrolled, was relying on volunteers to relay information. Although volunteers had a script to follow, the low enrolment rate may well have been a function of volunteers not contacting all residents about the study.

The limited sample size of this pilot study prevented more in-depth multivariable analysis and is not sufficient to rule out other important associations. Given the low proportion of eligible seniors who were enrolled, there may have been systematic differences between participants and non-participants that could have led to biased estimates of associations between exposures and outcomes.

Further research of larger populations is necessary to better understand the complex relationship between factors associated with acute care utilization in the elderly homecare population. It may also be important to test the potential benefits of targeted interventions such as dietary programs. Our experience demonstrates that although it may be feasible to conduct a large-scale study, major barriers, such as limited and possibly selective enrolment of participants resulting from the need to rely on agency staff, may hamper the success of such a study.

## Conclusion

In conclusion, the incidence of hospitalization and emergency department visits among seniors receiving homecare services is high. Presence of comorbidities appears to be an important predictor for hospitalization.

## Competing interests

The author(s) declare that they have no competing interests.

## Authors' contributions

AAS participated in acquisition of data, performed the statistical analysis, and assisted in drafting the manuscript. SBCC drafted the manuscript. KW participated in the design, coordination, and data interpretation of the study. TJB collected data. SDS participated in coordinating the study. TA participated in the design and interpretation of data. TM participated in the design and interpretation of data. ML conceived of the study, participated in its design and coordination, helped draft the manuscript and assisted in the analysis. All authors read and approved the final manuscript.

## Pre-publication history

The pre-publication history for this paper can be accessed here:


